# SMG8/SMG9 Heterodimer Loss Modulates SMG1 Kinase to Drive ATR Inhibitor Resistance

**DOI:** 10.1158/0008-5472.CAN-21-4339

**Published:** 2022-10-23

**Authors:** Marta J. Llorca-Cardenosa, Lauren I. Aronson, Dragomir B. Krastev, Jadwiga Nieminuszczy, John Alexander, Feifei Song, Malgorzata Dylewska, Ronan Broderick, Rachel Brough, Astrid Zimmermann, Frank T. Zenke, Bora Gurel, Ruth Riisnaes, Ana Ferreira, Theodoros Roumeliotis, Jyoti Choudhary, Stephen J. Pettitt, Johann de Bono, Andres Cervantes, Syed Haider, Wojciech Niedzwiedz, Christopher J. Lord, Irene Y. Chong

**Affiliations:** 1 The Institute of Cancer Research, London, United Kingdom.; 2 The CRUK Gene Function Laboratory, The Institute of Cancer Research, London, United Kingdom.; 3 Breast Cancer Now Toby Robins Breast Cancer Research Centre, The Institute of Cancer Research, London, United Kingdom.; 4 The healthcare business of Merck KGaA, Biopharma R&D, Translational Innovation Platform Oncology, Darmstadt, Germany.; 5 The Royal Marsden Hospital NHS Foundation Trust, London, United Kingdom.; 6 Department of Medical Oncology, INCLIVA Biomedical Research Institute, University of Valencia, Valencia, 46010, Spain.; 7 CIBERONC, Instituto de Salud Carlos III, Madrid, Spain.

## Abstract

Gastric cancer represents the third leading cause of global cancer mortality and an area of unmet clinical need. Drugs that target the DNA damage response, including ATR inhibitors (ATRi), have been proposed as novel targeted agents in gastric cancer. Here, we sought to evaluate the efficacy of ATRi in preclinical models of gastric cancer and to understand how ATRi resistance might emerge as a means to identify predictors of ATRi response. A positive selection genome-wide CRISPR-Cas9 screen identified candidate regulators of ATRi resistance in gastric cancer. Loss-of-function mutations in either SMG8 or SMG9 caused ATRi resistance by an SMG1-mediated mechanism. Although ATRi still impaired ATR/CHK1 signaling in SMG8/9-defective cells, other characteristic responses to ATRi exposure were not seen, such as changes in ATM/CHK2, γH2AX, phospho-RPA, or 53BP1 status or changes in the proportions of cells in S- or G_2_–M-phases of the cell cycle. Transcription/replication conflicts (TRC) elicited by ATRi exposure are a likely cause of ATRi sensitivity, and SMG8/9-defective cells exhibited a reduced level of ATRi-induced TRCs, which could contribute to ATRi resistance. These observations suggest ATRi elicits antitumor efficacy in gastric cancer but that drug resistance could emerge via alterations in the SMG8/9/1 pathway.

**Significance:**

These findings reveal how cancer cells acquire resistance to ATRi and identify pathways that could be targeted to enhance the overall effectiveness of these inhibitors.

## Introduction

Gastric cancer is the fifth most common cancer worldwide and the third leading cause of cancer-related death (1). Most patients relapse after curative resection and approximately 50% of patients present with advanced disease at the time of diagnosis. Despite the integration of therapeutic approaches that inhibit targets such as HER2 (2), VEGFR2 (3, 4), and immune checkpoints (5), the overall prognosis for patients with advanced disease remains poor.

Genetic alterations in the DNA damage response (DDR) pathway, which may confer sensitivity to DDR targeted therapies, are found in approximately a quarter of advanced gastric cancer (6). Ataxia-telangiectasia mutated and Rad3-related protein kinase (ATR), a member of the phosphoinositide 3 kinase–related kinase (PIKK) family, modulates the progression of DNA replication forks in S-phase, maintains genomic stability through the accurate replication of the genome and prevents premature mitotic entry, thus minimizing the transmission of damaged DNA and/or disordered genomes onto daughter cells (7–9). In particular, ATR allows cells to detect and manage replication fork stress (RFS), as well as the slowing or stalling of replication fork progression and/or DNA synthesis (10). ATR responds to this and other forms of RFS by suppressing further transcription-replication conflicts (TRC), promoting replication fork recovery, enforcing G_2_–M cell-cycle arrest (to allow replication to be restarted and completed prior to mitosis) and preventing excessive cleavage of reversed forks (11, 12).

Tumor cells often exhibit a greater reliance upon ATR function than normal cells, a phenotype that can be exploited via small molecules, ATR inhibitors (ATRi), that inhibit ATR kinase activity (13). Preclinical work has established that defects in *ATM* or *ARID1A* cause ATRi sensitivity (14–16). Although *ATM* mutations are present in 5% of primary gastric cancers, loss of ATM protein expression has been reported to occur in 17% of the cases (6, 17–19), while truncating mutations in *ARID1A* are present in approximately 20% of gastric cancer (6, 18, 19), which supports the case for the clinical assessment of ATRi in gastric cancer in appropriately stratified populations. Furthermore, ATRi sensitivity has been seen in an ATM-defective gastric cancer tumor cell line (20) and in organoid models of gastric cancer (21).

When assessed in non–gastric cancer–focused early phase clinical trials, ATRi such as ceralasertib (AZD6738), berzosertib (M6620, VX-970) or elimusertib (BAY1895344) can elicit significant antitumor effects (22–29), including profound responses in prostate, breast, endometrial, renal, or appendiceal tumors, some of which have ATM or ARID1A defects (30, 31). Understanding how ATRi resistance might emerge in gastric cancer could also help refine biomarkers of drug resistance for patient treatment stratification. The mechanism by which ATRi resistance in gastric cancer occurs is currently unknown. However, a CRISPR-Cas9 genetic perturbation screen in a non–gastric cancer cell line has shown that loss of *CDC25A*, a mitosis-promoting factor that normally promotes mitotic entry by eliminating WEE1-mediated inhibitory phosphorylation on CDK1, causes ATRi resistance (32).

SMG8 and SMG9, members of the suppressors with morphologic effects on genitalia (SMG) protein family, form a heterodimer with a canonical role in controlling nonsense-mediated mRNA decay (NMD; ref. 33), a process that prevents the accumulation of truncated proteins in the cell (34, 35). A key event in the triggering of NMD is phosphorylation of the RNA helicase, UPF1, by another member of the SURF (SMG1-UPF1-eRF1-eRF3) complex, the phosphoinositide 3 kinase–related kinase (PIKK), SMG1 (36). UPF1 phosphorylation results in 3′ mRNA unwinding and the recruitment of the decay-inducing factors SMG5, SMG6, and SMG7 (37). In part, the activation of UPF1 by SMG1 is controlled by SMG8/9, which suppresses SMG1 activity (33, 38–40). In addition to its role in NMD, SMG1 has also been implicated in the DDR. For example, silencing of SMG1 causes constitutive phosphorylation of CHK2 and p53 (41), the formation of γH2AX foci and an increase in the number of chromosomal aberrations in human tumor cell lines (42, 43). SMG1, UPF1, SMG6, SMG7, and other NMD proteins (which undertake their NMD-related roles in the cytoplasm), have additionally been detected in the nucleus (41, 42, 44, 45). NMD proteins are also thought to play a nuclear role in telomere stability by negatively regulating telomeric repeat-containing noncoding RNA (TERRA; ref. 46).

Here, we used a positive selection genome-wide CRISPR-Cas9 screen to identify candidate regulators of ATRi resistance in gastric cancer, including loss-of-function mutations in either *SMG8* or *SMG9* that appeared to cause ATRi resistance by a SMG1-mediated mechanism. We subsequently found that although ATRi still impair ATR/CHK1 signaling in SMG8/9-defective cells, many of the other characteristic responses to ATRi exposure (ATM/CHK2, γH2AX, phospho-RPA and 53BP1 responses, S-phase, and G_2_–M cell-cycle alterations) were not present. Moreover, loss of SMG8/9 reduced the burden of ATRi-elicited transcription/replication conflicts (TRC) in cells, suggesting a way SMG8/9 defects contribute to ATRi resistance.

## Materials and Methods

An extended version of the methodology, as well as the tables containing all the reagents used in this study (Supplementary Table S1) and antibodies (Supplementary Table S2) can be found in the Supplementary Data section.

### Cell lines

AGS, HEK239T, HCT116, and NCIN87 (ATCC); SNU1, SNU5, SNU484, and SNU638 (Korean Cell Line Bank); YCC6 (gift from Professor Sun Young RHA, Yonsei Cancer Centre, South Korea) and HAP1 cells (Horizon Discoveries) were maintained as per the supplier's instructions. Cell line identity and *Mycoplasma* infection was tested periodically by using short tandem repeat typing StemElite Kit (Promega) and MycoAlert *Mycoplasma* Detection Kit (Lonza), respectively.

### Cellular viability assays

Cells were seeded in 384-well plates at an approximate number of 500 cells per well. Drug was added 24 hours after seeding and plates were incubated at 37°C for 5 days. Viability was estimated using CellTiter-Glo luminescence reagent (Promega). Final fluorescence intensity value was normalized to DMSO median and surviving fractions of cells were plotted where lines of best fit were drawn using a four-parameter nonlinear regression. Surviving fraction 50 (SF_50_), the concentration of drug required to cause a 50% inhibition of the cell population, or AUC values were calculated from these curves using GraphPad Prism software. Comparisons of dose–response curves were performed using two-way ANOVA testing. Comparisons of SF_50_ or AUC data were performed using the Mann–Whitney test for nonparametric samples. Results represent the mean of at least three independent experiments.

### siRNA transfection knockdown experiments

Reverse transfections using the siRNA SMARTpool, siCON1 and siCON2 negative controls (Dharmacon) were carried out in 384-well plates, 6-well plates or 10 cm dishes using 20 nmol/L of siRNA (unless specified), mixed with 12.5% of the final volume of optiMEM and incubated at room temperature for 10 minutes. In parallel, 20 nmol/L RNAiMax (Thermo Fisher Scientific) was added to 12.5% of the final volume of optiMEM and incubated at room temperature for 10 minutes. siRNA and RNAiMax mixtures were mixed and incubated at room temperature for 30 minutes before applying to the cells. Lysates were retrieved or viability experiments were performed after 2 to 3 days.

All viability assays were performed in triplicate using CellTiter-Glo luminescence reagent (Promega). The surviving fraction was calculated as follows: Surviving fraction = (luminescence in siRNA treated well)/(luminescence in siControl treated wells). The normalized percentage of inhibition, to normalize data between different cell lines by the efficiency of transfection, was calculated using the following formula: ((mean (positive control) − Sample)/(mean (positive control) − mean (negative control)) × 100. Supplementary Table S1 contains all the siRNAs used in this study.

### Cell cycle

For cell-cycle analysis, cells were seeded in 6-well plates at a 60% confluency and treated the following day with 150 nmol/L berzosertib or DMSO. After 48 hours, cells were stained for 1 hour with 20 μmol/L 5-ethynyl-2′-deoxyuridine (EdU). Cells were then harvested and fixed with ice-cold 70% ethanol overnight. Cell-cycle distribution was assessed using the Click-IT EdU kit (Thermo Fisher Scientific) with Alexa647. RNA was removed by digestion with RNAse A (Sigma-Aldrich) for 30 minutes at 37°C, before propidium iodide (Sigma-Aldrich) was added to the cells. Data were acquired on a BD LSR II flow cytometer (BD Biosciences). Debris and doublets were gated out from a Forward scatter/Side scatter dot plot and DNA dye area/width dot plot, respectively and the selected population was analyzed regarding its cell distribution using the FACS diva software.

### Positive selection genome-wide CRISPR/Cas9

Doxycycline-inducible Cas9-expressing cells were generated by transduction of YCC6 cells with the Edit-R Inducible Lentiviral hEF1a-Blast-Cas9 Nuclease (Dharmacon) and selected in 7 μg/mL blasticidin for 5 days (YCC6^iCas9^). Cas9 catalytic activity was tested using a dual-fluorescence protocol, transducing cells with a GFP/red fluorescent protein (RFP)-expressing construct (GFP/RFP/empty), or with the same construct carrying an additional single-guide RNA (sgRNA) sequence toward GFP protein (GFP/RFP/gfp-sgRNA). Cells were then treated with doxycycline for at least 2 days, retrieved, and green and red fluorescence was analyzed by flow cytometry using the BD LSRII (Beckton Dickinson).

For the screen, YCC6^iCas9^ cells were seeded aiming for 1,000× representation per sgRNA in the library, and infected at a multiplicity of infection (MOI) of 0.3, to avoid multiple sgRNA infections per cell, with a previously published and validated genome-wide human lentiviral CRISPR library (47). Efficiently transduced cells were selected with 5 μg/mL puromycin for 5 days, when a sample T = 0 was taken (<1,000× sgRNA representation number of cells). After the T = 0 sample was taken, 1 million cells were plated in 15-cm plates, maintaining the 1,000× sgRNA representation, and 100 nmol/L berzosertib (SF0) was added to the cells. Growth media was changed and cells were drugged twice a week for 3 weeks, before T = 1 was taken. DNA was extracted from samples T = 0 and T = 1 and PCR of the CRISPR guide regions were carried out. sgRNA in each sample were sequenced using a U6 custom primer (Supplementary Table S1) on the HiSeq (Illumina) to generate gRNA count data (all the information about the screen analysis can be found in Supplementary Materials and Methods section).

### Arrayed focused validation screen

An initial validation of the hits was carried out in a 96-well format using an arrayed CRISPR/Cas9 reaction with individual guides in each well. Each gene was targeted using five or more parallel crRNA and two negative control sgRNAs (with no homology toward any human gene) were included on each plate. In each well, 1,500 YCC6^iCas9^ cells were reversely transfected with 5 μL of 2 μmol/L sgRNA and 5 μL of 2 μmol/L tracrRNA in 20 μL of OptiMEM, using 3.5 μL of 1:10 diluted RNAiMAX (Thermo Fisher Scientific), and incubated for 24 hours. The following day, media was removed and fresh media, containing 80 nmol/L berzosertib was added (a concentration high enough to kill all YCC6 cells in normal conditions). CellTiter-Glo luminescence reagent (Promega) was used to measure the number of living cells in each well, and these results were compared with the negative control cells to determine which crRNAs caused resistance to the lethal doses of berzosertib.

### SMG8 cDNA expression

*SMG8* or *GFP* cDNA (EZShuttle Gateway Plus vector, Labomics) were cloned into a HA-tagged pInducer20 plasmid (Addgene), using Gateway LRclonase enzyme mix (Thermo Fisher Scientific) as per manufacturer's instructions. Lentiviral particles containing GFP or tagged SMG8 were produced in HEK293T cells as described previously (48) and used to transduce the cells in 600 μg/mL G418 selective media.

### Immunofluorescence and DNA fibre assays

For 53BP1 and γH2AX immunofluorescence experiments, cells were seeded on coverslips the day before, so they reached 60% confluency when exposing them to varying concentrations of ATRi berzosertib or siRNA transfection. After treatment, cells were fixed using 4% paraformaldehyde for 15 minutes, washed twice in PBS and permeabilized in 0.2% Triton-X-100 for 10 minutes at room temperature. After washing three times in PBS, cells were blocked in 10% FBS in PBS for 30 minutes and incubated with the corresponding primary antibody for 1 hour at room temperature in a humidifier chamber. After four washes in PBS, cells were incubated with secondary fluorescent antibody for 45 minutes and washed four times before mounting them onto slides using DAPI-containing Vectashield medium (Vector Lab).

For the detection of R loops, cells were grown on coverslips overnight and then washed with PBS, fixed in ice-cold methanol for 10 minutes, permeabilized with ice-cold acetone for 1 minute, washed with PBS and blocked for 1 hour at room temperature in 3% BSA and 0.1% Tween 20 in 4x SSC buffer. For primary immunolabeling, cells were incubated in blocking buffer with S9.6 antibody (1:500; mouse, Kerafast) for 3 hours at room temperature. Cells were then washed three times with PBS followed by incubation with AlexaFluor 555–conjugated secondary antibody (1:500, Thermo Fisher Scientific) in blocking buffer for 1 hour at room temperature followed by three washes of PBS. For our RNASEH1 controls, ATRi exposed coverslips were incubated with RNASEH1 solution (5 units/100 μL solvent per coverslip) for 1–2 hours at 37 degrees, prior to blocking. Images were acquired using Leica SP8 laser scanning confocal microscope with LasX software on 63× objective.

Proximity ligation assays (PLA) were conducted as described previously (12, 49, 50), by seeding cells in coverslips at a concentration of 200,000 cells/mL 24 hours before DMSO, ATRi (300 nmol/L, 24 hours), DRB (80 μmol/L, 2 hours), or XL413 (5 μmol/L, 4 hours) exposure. More than 100 cells per condition were analyzed, in a total of three or more biological repeats. Cells were analyzed by measuring nuclear staining in the case of γH2AX and S9.6 (R loops), whereas 53BP1 or PLA foci were scored counting the number of foci per nucleus using FIJI (ImageJ) software.

DNA fibre assays were performed using the method widely explained in ref. 51. *P* values were calculated using Mann–Whitney test for nonparametric samples, measuring sister fork length ratio (a.u.) using FIJI (ImageJ).

### Statistical analysis

Statistical analysis was performed using GraphPad Prism. All tests were two sided unless otherwise stated. Mann–Whitney tests were used to compare nonparametric datasets and Student *t* tests used for parametric datasets.

### Data availability

The data generated in this study are available within the article and its Supplementary Data.

## Results

### Gastric cancer genome-wide CRISPRn screen identifies genetic determinants of ATRi resistance

**Figure 1.**
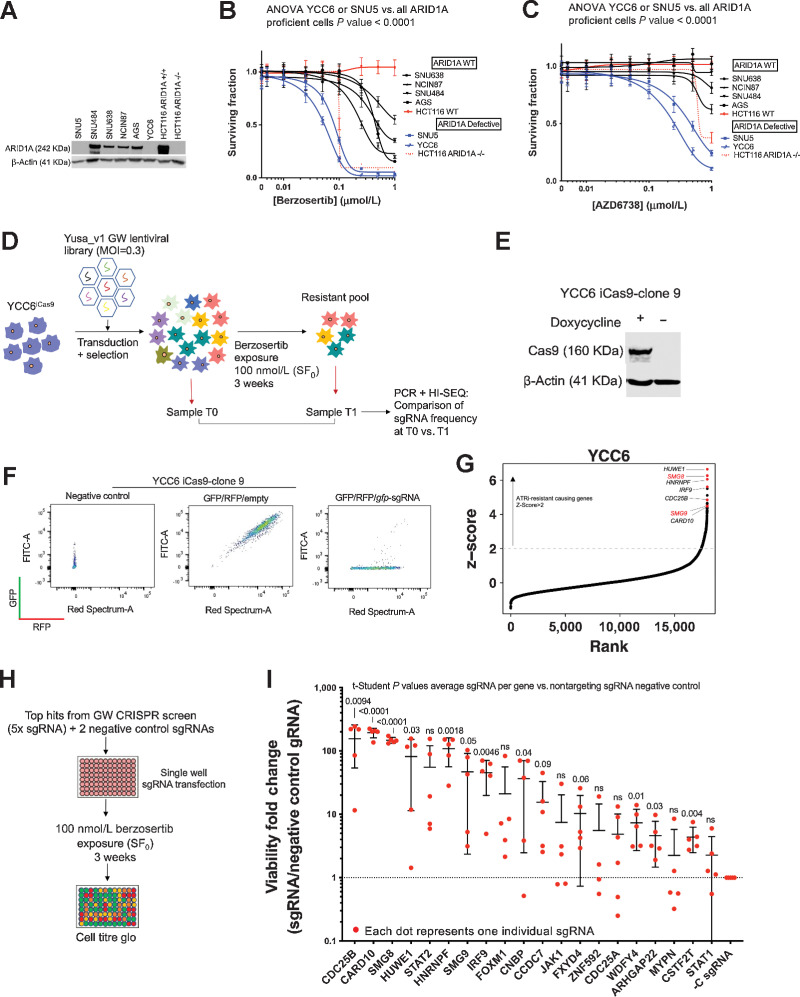
Gastric cancer genome-wide CRISPRn screen identifies genetic determinants of ATRi resistance. **A,** YCC6 and SNU5 gastric tumor cell lines show no ARID1A expression by Western blotting. Other ARID1A-proficient gastric tumor cell lines are represented in this Western blot analysis and HCT116 ARID1A isogenic cell lines were used as positive and negative controls. **B** and **C,** Dose–response survival curves (384-well plate, 5-day assay) show that YCC6 and SNU5 (blue) are sensitive to ATRi, compared with the ARID1A-proficient gastric cancer cell lines (black). HCT116 isogenic controls are highlighted in red. **D,** Schematic illustrating a genome-wide ATRi CRISPR/Cas9 screen using the YCC6 gastric cancer cell line. **E,** Western blot analysis showing that YCC6 gastric tumor cell line (clone 9) expresses Cas9 upon doxycycline induction after being transduced with an Edit-R Inducible Lentiviral hEF1a-Blast-Cas9 Nuclease vector and selected with blasticidin. **F,** YCC6 iCas9 cells have a catalytically active Cas9 as shown by flow cytometry. GFP/RFP/Empty represent iCas9 cells transduced with GFP- and RFP-expressing lentiviral constructs. GFP/RFP/gfp-sgRNA represents iCas9 cells that were additionally transduced with a sgRNA-targeting GFP that cleaves the GFP protein thereby decreasing green fluorescence emission. **G,** Scatter plot illustrating sgRNA z-score for ATRi-resistant cells retrieved at T1 compared with untreated cells retrieved at T0 plotted against the rank calculated from the rank product of z-score and MAGeCK analysis of sgRNA counts. The genes targeted by sgRNA that were most enriched (T1-T0) are highlighted in red at the top right corner of the graph comprising *CDC25B*, *SMG8*, *SMG9*, *HUWE1*, *IRF9*, *HNRNPF*, and *CARD10*. **H,** Diagram showing the validation screen workflow. **I,** Results of the deconvoluted CRISPRn validation screen in 96-well plate format. Each red dot represents an individual sgRNA. *P* values were calculated by conducting a *t* test comparing all sgRNAs per gene versus all sgRNAs per the control sgRNA. Dose–response curves, Western blot analysis, and the deconvoluted CRISPRn validation screen in 96-well plate format are representative of three or more biological replicates. ns, nonsignificant.

Prior reports have suggested that gastric cancer tumor cell lines or gastric cancer tumor organoids exhibit ATRi sensitivity (20, 21). We confirmed these observations in seven gastric cancer patient-derived xenografts (PDX), using M4344, an orally bioavailable ATRi (NCT04655183). This analysis indicated that short periods of M4344 treatment were sufficient to elicit antitumor gastric cancer PDX responses, especially in PDX with either ARID1A or ATM defects (Supplementary Fig. S1A–S1N). In a complementary panel of gastric cancer tumor cell lines, we found that two cell lines with ARID1A defects (YCC6 and SNU5) showed profound sensitivity to two different small-molecule ATRi, berzosertib and AZD6738. The level of ATRi sensitivity in YCC6 and SNU5 cells was comparable with that seen in previously validated *ARID1A* mutant HCT116 isogenic cell pair (Fig. 1A–C; ref. 52), allowing us to select YCC6 tumor cells as a “ATRi sensitive” cell line for use in a later CRISPR-Cas9 mutagenesis screen for determinants of ATRi resistance.

To identify mechanisms of ATRi resistance in gastric cancer, we then carried out a positive selection GW CRISPR-Cas9 mutagenesis screen in YCC6 cells, using a human sgRNA library encompassing 87,897 sgRNAs (Fig. 1D; ref. 47). We first generated and validated doxycycline-inducible Cas9-expressing YCC6 cells (YCC6^cas9^; Fig. 1E and F), transduced these with the sgRNA library at a low MOI (MOI = 0.3) and after activating Cas9, exposed these to a lethal concentration of berzosertib [surviving fraction = 0 (SF_0_); 100 nmol/L] for 3 weeks. By using deep sequencing to compare sgRNA frequencies in pre-ATRi and post-ATRi cell populations, we identified a list of candidate determinants of ATRi resistance (Fig. 1G; Supplementary Table S3). To evaluate the sensitivity of the screen, we undertook an analysis describing the performance of guide RNAs targeting “core essential” genes, a commonly used metric to determine the quality of genetic perturbation screens (Supplementary Fig. S1O). This analysis detected a statistically significant depletion of sgRNAs targeting commonly accepted core essential genes, suggesting our screen was of high quality. We also compared our screen data with other published CRISPR-Cas9 screens for ATRi resistance (53–55) and found substantial overlap between our screen and those of others (Supplementary Fig. S1P–S1R). Although it is expected that there will be some hits that are private to the model systems used, 20% of the ATRi resistance–causing hits in our screen were also seen in comparable screens carried out in different tumor cell lines (Supplementary Fig. S1P–S1R). Finally, to ensure our analysis was not wholly influenced by an individual screen analysis method, we also analyzed the screen data using two different methods, Z-score and MAGeCK, thus identifying robust ATRi resistance–causing effects (Supplementary Table S3).

Some of the most profound effects we observed included those caused by CRISPR-Cas9 targeting of *CDC25B*, *SMG8*, *SMG9*, *HUWE1*, *HNRNPF*, *IRF9*, or *CARD10* (Fig. 1G). Using a subsequent 96-well plate validation screen, YCC6^cas9^ cells transfected with one sgRNA/well, using a library of five different sgRNAs/gene (Fig. 1H; ref. 47), we confirmed the ATRi resistance–causing effects of 13 genes including *CDC25B* (Fig. 1I), a homolog of the known modulator of ATRi resistance *CDC25A* (32). Consistent with the literature, one of the sgRNA designed to target *FOXM1*, a critical proliferation-associated transcription factor (56), was also validated as a mediator of ATRi resistance; FOXM1 phosphorylation is known to be tightly controlled by ATR and reduced FOXM1 levels have been shown to promote the completion of DNA replication, preventing ATRi-induced premature mitosis and subsequent genome instability (57). Among the most profound ATRi resistance–causing effects were those caused by sgRNA targeting SMG8 or SMG9 (Fig. 1I). SMG8 and SMG9 form a heterodimer whose canonical role is in suppressing the NMD functions of the kinase SMG1 (see Introduction).

To assess whether the effects of SMG8/9 sgRNA were private to YCC6 cells, we reanalyzed previously described CRISPR-Cas9 screens involving ATRi resistance (55), finding that sgRNA-targeting SMG8 caused ATRi resistance in MCF10A (human nontumor mammary epithelial cells), HCT116 (human colorectal tumor cell line), and HEK293A cells (human embryonic kidney); and SMG9 sgRNA caused ATRi resistance in HEK293A cells (Supplementary Fig. S1P–S1R). This suggested that ATRi resistance mediated by SMG8 or SMG9 loss of function might be a more generalizable effect and not private to YCC6- or ARID1A-defective cells.

### SMG8/9 deficiency causes resistance to ATRi via a SMG1-dependent process

**Figure 2.**
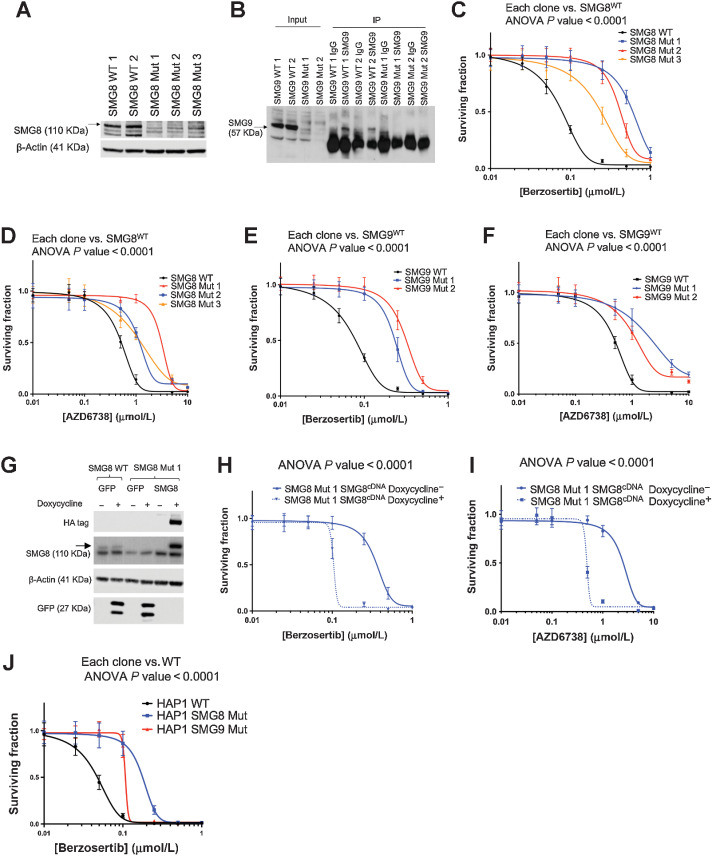
SMG8 and SMG9 deficiency causes resistance to ATR inhibition. **A,** YCC6 SMG8 mutant clones show lower levels of SMG8 protein expression compared with SMG8 WT YCC6 cells by Western blotting. **B,** YCC6 SMG9 mutant clones show no SMG9 protein expression compared with SMG9 WT YCC6 cells by immunoprecipitation. **C–F,** SMG8 and SMG9 mutant clones are resistant to ATRi (384-well plate, 5-day assay) compared with the WT cells. **G,** Western blot analysis showing doxycycline-inducible overexpression of HA-tagged SMG8 cDNA in SMG8 Mut 1 clone. Doxycycline-inducible GFP cDNA overexpression served as a negative control. **H** and **I,** ATRi dose–response survival curves (384-well plate, 5-day assay) illustrating a resensitization to berzosertib and AZD6738 in the SMG8 Mut 1 clone when SMG8 overexpression was induced by doxycycline exposure (dotted line, compared with the continuous line). **J,** SMG8 (blue) and SMG9 (red) mutant HAP1 cells are resistant to ATRi (384-well plate, 5-day assay). All panels of this figure are representative of three or more biological replicates.

To evaluate the mechanism by which SMG8 and SMG9 defects cause ATRi resistance, we used CRISPR-Cas9 mutagenesis to create SMG8 or SMG9 mutant YCC6 daughter clones (hereafter termed SMG8 Mut 1, 2, or 3 and SMG9 Mut 1 or 2). SMG8 Mut 1 cells harbored a homozygous *SMG8* premature truncating mutation and loss of wild-type (WT) protein expression, whereas SMG8 Mut 2 and Mut 3 clones both possessed compound heterozygous truncating mutations and loss of WT protein expression (Fig. 2A; Supplementary Fig. S2A). SMG9 Mut 1 cells harbored a homozygous *SMG9* premature truncating mutation and loss of WT protein expression, whereas SMG9 Mut 2 cells possessed a heterozygous truncating mutation, an in-frame deletion and loss of WT protein expression (Fig. 2B; Supplementary Fig. S2B). Each SMG8 or SMG9 Mut clone exhibited significant resistance to AZD6738 or berzosertib, when compared with parental WT cells (Fig. 2C–F). We were also able to restore ATRi sensitivity in SMG8 Mut 1 by expressing WT, HA epitope–tagged, SMG8 cDNA (Fig. 2G–I), establishing a causal relationship between SMG8 dysfunction and ATRi resistance (Fig. 2H and I; Supplementary Fig. S2C and S2D). ATRi resistance within the context of SMG8 or SMG9 deficiency was not unique to the YCC6 cells; SMG8 or SMG9 mutant HAP1 cells (chronic myelogenous leukemia cells with homozygous premature truncating mutations and loss of WT protein; Supplementary Fig. S2E–S2H) were also resistant to ATRi (Fig. 2J; Supplementary Fig. S2I). Furthermore, siRNA gene silencing of SMG8 or SMG9 caused ATRi resistance in HCT116 cells (Supplementary Fig. S2J–S2O), consistent with our prior reanalysis of CRISPR-Cas9 screens (Supplementary Fig. S1P; ref. 55).

**Figure 3.**
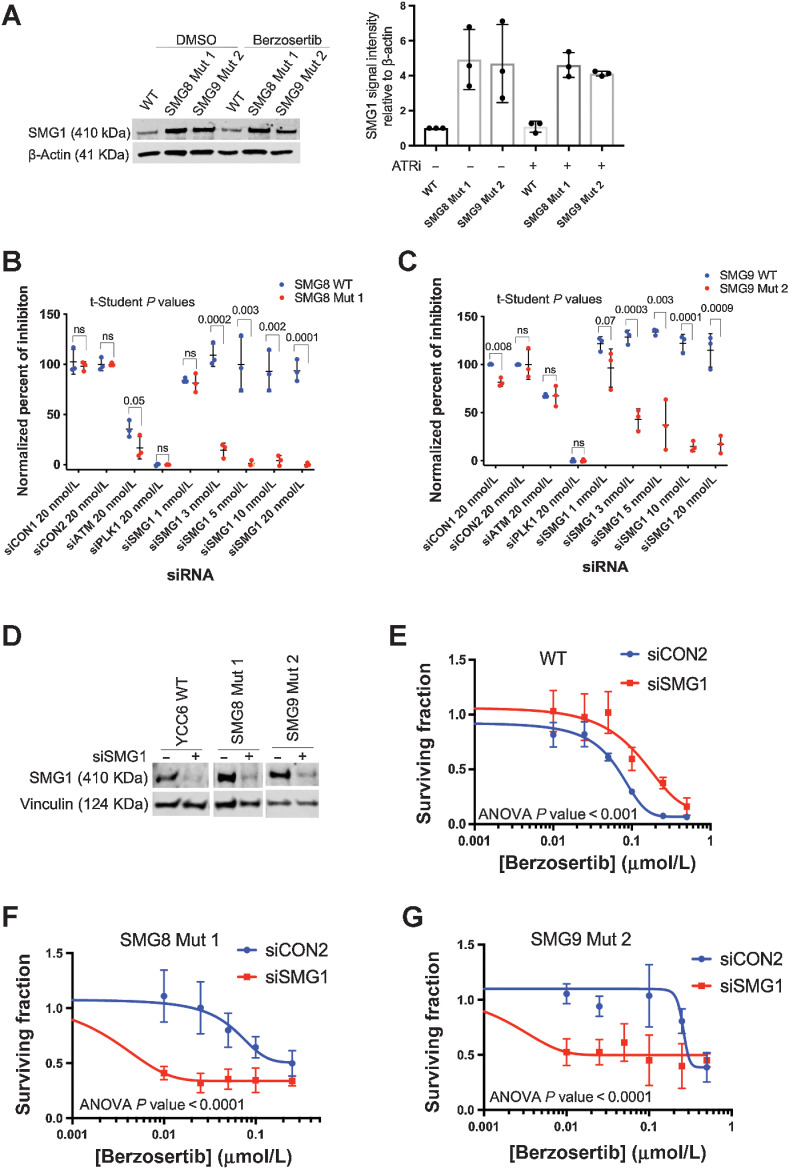
Silencing of SMG1 resensitizes SMG8 and SMG9 knockout cells to ATR inhibition. **A,** Left, SMG1 was overexpressed in the SMG8 and SMG9 mutant cells after 48 hours of DMSO or 150 nmol/L berzosertib exposure compared with the WT cells. Right, Smg1 signal intensity relative to β-actin expression representing relative protein expression using ImageJ. Error bars represent SEM, considering three biological replicates. **B** and **C,** SMG1 knockdown experiment (384-well plates) showing an increase in the normalized percent of inhibition in the SMG8 and SMG9 mutant cells (red) compared with the WT cells (blue) following exposure to a range of SMG1 siRNA concentrations (from 1 to 20 nmol/L; negative controls, siCON1, siCON2; positive controls, siATM, siPLK1). **D,** Western blot analysis showing siSMG1 silencing in the YCC6 WT and SMG8 and SMG9 mutant cells. **E–G,** Silencing of SMG1 in YCC6 WT, SMG8, and SMG9 mutant cells resensitizes them to ATRi (384-well plate, 5-day assay). siCON2 was used as a negative control. All panels of this figure are representative of three or more biological replicates. ns, nonsignificant.

SMG8 and SMG9 are known to negatively regulate SMG1 kinase activity (33, 38, 39). We noted that SMG1 protein expression (either in the presence or absence of ATRi) was elevated in YCC6 SMG8 Mut 1 and SMG9 Mut 2 cells (Fig. 3A). Because validating the causative role of SMG1 in ATRi resistance via cDNA expression of SMG1 was not possible due to the large size of the SMG1 coding sequence (10.98 kb), we used SMG1 siRNA to test whether the ATRi resistance in SMG8 or SMG9 mutant cells was SMG1-dependent. Initially, we found that SMG1 siRNA (in the absence of ATRi) inhibited SMG8 or SMG9 mutant cells more than SMG8/9 WT cells (Fig. 3B and C), suggesting that SMG8/9 mutant cells had become addicted to SMG1, an effect confirmed using multiple individual siRNAs (Supplementary Fig. S3A and S3B). Using the lowest concentration of SMG1 siRNA that elicited detectable gene silencing without causing detectable cell inhibition (1 nmol/L; Fig. 3D), we found that SMG1 silencing caused a profound resensitization to ATRi in SMG8 or SMG9 mutant cells, but caused a modest increase in ATRi resistance in SMG8/9 WT cells (Fig. 3E–G), suggesting that ATRi resistance in SMG8 or SMG9 mutant cells was a SMG1-dependent effect and likely related to the role SMG8/9 plays in suppressing SMG1 function.

Independently of its role in NMD, SMG1 has been described to play a role in DDR, possibly via SMG1 phosphorylation of the helicase UPF1 (38, 42, 58–61). UPF1 has previously been described to localize to the chromatin-bound fraction, where it exerts DNA replication and repair-related functions (34, 38–42). As expected, we observed a mild increase in UPF1 phosphorylation in SMG8 and SMG9 mutant cells (Supplementary Fig. S3C). Interestingly, UPF1 siRNA increased ATRi resistance in SMG8/9 WT cells but not in SMG8 or SMG9 mutant cells (Supplementary Fig. S3D–S3F), an effect confirmed using multiple individual siRNAs (Supplementary Fig. S3G–S3I), suggesting a preexisting UPF1 defect in SMG8/9 mutant cells that could lead to ATRi resistance.

### SMG8/9 mutations do not reverse the inhibition of ATR/CHK1 signaling but abrogate the cell-cycle effects of ATR inhibition

**Figure 4.**
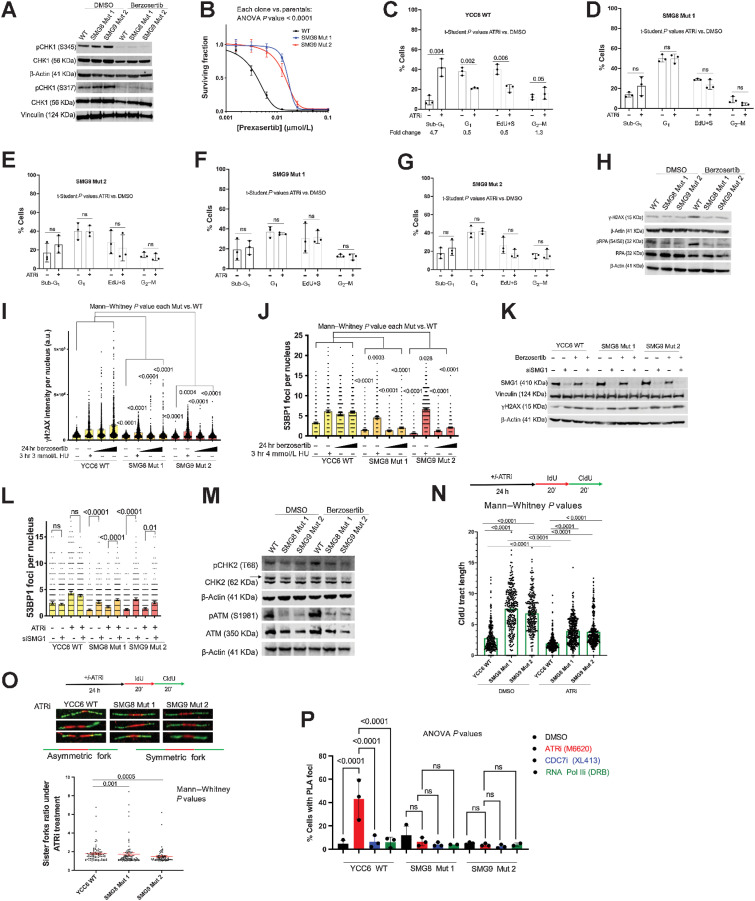
SMG8/9 mutation does not reverse the inhibition of ATR/CHK1 signaling but abrogates ATRi-associated cell-cycle effects, RFS, and TRCs. **A,** SMG8 and SMG9 mutant cells do not show increased pCHK1 (Ser317 and Ser345) or total CHK1 protein expression levels compared with the WT cells after 24 hours of DMSO or 400 nmol/L berzosertib exposure. **B,** SMG8 (blue) and SMG9 (red) mutant cells are resistant to the CHK1i prexasertib (384-well plate, 5-day assay) compared with the WT cells (black). **C–G,** Flow cytometry EdU/propidium iodide staining cell-cycle analysis showing the percentage of cells in sub-G_1_, G_1_, active S-phase (EdU+ cells), and G_2_–M cells in the WT cells, two SMG8 mutant and two SMG9 mutant clones after 48 hours of DMSO or 150 nmol/L ATRi exposure. **H,** SMG8 and SMG9 mutant cells show lower levels of γH2AX and pRPA (S4/S8) protein expression after 48 hours of DMSO or 150 nmol/L berzosertib exposure by Western blot analysis. **I,** SMG8 (orange) and SMG9 (red) mutant cells show lower levels of γH2AX intensity compared with the WT cells (yellow) after 24 hours of DMSO or 400 or 800 nmol/L of ATRi exposure, despite showing an increase in γH2AX intensity after 3 hours of 3 mmol/L HU. A complete version of the representative image can be found in Supplementary Fig. S4D and S4E. **J,** SMG8 (orange) and SMG9 (red) mutant cells show lower levels of p53BP1 foci compared with the WT cells (yellow) after 24 hours of DMSO or 400 or 800 nmol/L of ATRi exposure, despite showing an increase in the number of 53BP1 foci after 3 hours of 3 mmol/L HU. A complete version of the representative image can be found in Supplementary Fig. S4D and S4E. **K,** Western blot analysis showing that SMG1 knockdown rescues levels of γH2AX protein expression in the SMG8 and SMG9 mutant cells after 24 hours of DMSO or 300 mmol/L berzosertib exposure in cells transfected with 1 nmol/L siSMG1 or siCON2, 72 hours before protein extraction. **L,** SMG1 knockdown rescues the levels of 53BP1 foci in the SMG8 and SMG9 mutant cells after 24 hours of DMSO or 400 or 800 nmol/L of berzosertib exposure after their transfection with 2 μmol/L of siSMG1 or siCON2, 72 hours before protein extraction. HU (3 hours of 3 mmol/L) was used as a positive control. **M,** SMG8 and SMG9 mutant cells show lower levels of pCHK2 (T68) and pATM (S1981) protein expression by Western blotting in the SMG8 Mut 1 and SMG9 Mut 2 cells after 48 hours exposure to DMSO or 150 nmol/L berzosertib compared with the WT. **N,** SMG8 and SMG9 mutant cells show increased CIdU track length (a.u.) compared with WT cells in a DNA fiber assay after 24 hours of 300 nmol/L berzosertib or DMSO exposure (20 minutes incubation with 25 μmol/L IdU and 20 minutes incubation with 125 μmol/L CIdU). **O,** SMG8 and SMG9 mutant cells present decreased forks ratio (a.u.) compared with the WT cells in a DNA fiber assay after 24 hours of 300 nmol/L berzosertib exposure (20 minutes incubation with 25 μmol/L IdU and 20 minutes incubation with 125 μmol/L CIdU). **P,** SMG8 and SMG9 mutant cells present lower levels of RNAPolII/PCNA PLA foci (measuring TRCs) per cell after 24 hours of 300 nmol/L ATRi, DMSO, or 2 hours of 80 μmol/L of the RNAPII inhibitor DRB exposure. *P* values were calculated using a one-way ANOVA test). All images are representative of three or more biological replicates. ns, nonsignificant.

Although we had established that the ATRi resistance phenotype in SMG8/9-defective cells was a SMG1-mediated effect, it was not clear whether *SMG8* or *SMG9* mutation caused resistance by restoring ATR function, by minimizing the effects of ATRi on the genome and replication forks and/or caused ATRi resistance by preventing premature mitotic entry, as is the case for ATRi resistance caused by loss of CDC25A (32). To assess whether ATR function had been restored in SMG8/9-defective cells, we measured levels of phosphorylated CHK1 (Ser345 and Ser317) in cells exposed to ATRi. Although berzosertib exposure reduced CHK1 Ser^345^ and Ser^317^ phosphorylation, this was not reversed in SMG8/9 mutant cells (Fig. 4A). Furthermore, we found that SMG8/9 mutant cells were resistant to the CHK1 small-molecule inhibitor, prexasertib (Fig. 4B), suggesting that the ATRi resistance seen in these models was not a direct result of the restoration of ATR-CHK1 function.

Previous work has demonstrated that loss of CDC25A causes ATRi resistance by preventing premature mitotic entry and eventual mitotic catastrophe, effects that can be reversed by WEE1 inhibition (32). SMG1 has also previously been implicated in CDC25A control (43), raising the possibility that increased SMG1 activity caused by loss of SMG8/9 could cause ATRi resistance via CDC25A modulation and imposition of G_2_–M cell-cycle arrest. Using flow cytometry cell-cycle analysis, we found that (as expected) exposure of SMG8/9 WT cell to berzosertib caused an increase in the sub-G_1_ fraction, a reduction in active S-phase cells (EdU+ cells) and invocation of the G_2_–M cell-cycle checkpoint (Fig. 4C). Each of these ATRi-induced effects was reversed in SMG8 or SMG9 mutant cells (Fig. 4D–G), suggesting that *SMG8* or *SMG9* mutation allows cells to progress efficiently through the cell cycle in the face of ATRi exposure, and that the control of the cell cycle via CDC25A loss was unlikely to explain ATRi resistance in this case. Furthermore, SMG8 and SMG9 Mut clones did not express higher levels of CDC25A or CDC25B (Supplementary Fig. S4A) and exposure to the WEE1 inhibitor (AZD1775, WEE1i), that is able to restore premature mitotic entry in the face of CDC25A loss (32), had identical effects on ATRi sensitivity in SMG8/9 WT and mutant cells (Supplementary Fig. S4B and S4C). This indicated that CDC25A is unlikely to be a key mediator of ATRi resistance in SMG8/9 mutant cells.

### SMG8/9 mutations prevent RFS and TRCs

Given that *SMG8/9* mutations prevented ATRi from causing their expected effects on the cell cycle, we assessed whether ATRi elicited activation of the DDR in the absence of WT SMG8/9. The RFS that ATRi cause often results in: (i) sustained phosphorylation of the single-strand binding protein RPA (pRPA); (ii) phosphorylation of histone H2AX (γH2AX); and (iii) the formation of nuclear 53BP1 foci (32). Using Western blotting, we found that SMG8 or SMG9 mutant cells displayed lower levels of γH2AX and pRPA following ATRi exposure (Fig. 4H). Using immunofluorescence and confocal microscopy, we found that SMG8 or SMG9 mutant cells also mounted significantly reduced γH2AX and 53BP1 focus formation after ATRi exposure (Fig. 4I and J; Supplementary Fig. S4D and S4E). We noted that the γH2AX and 53BP1 responses in SMG8/9 mutant cells were restored to those seen in SMG8/9 WT cells when we transfected cells with SMG1 siRNA (Fig. 4K and L). Interestingly, the γH2AX and 53BP1 responses to hydroxyurea (HU), an agent that causes RFS by depleting deoxynucleotide triphosphates (62), were not blunted in SMG8/9 mutant cells to the same extent as when cells were exposed to ATRi (Fig. 4I and J; Supplementary Fig. S4D and S4E). This suggested that SMG8/9 mutant cells were able to respond to perturbations that impair replication fork progression but not to the specific types of cellular stress that ATRi cause. One way cells buffer ATR inhibition is through the activation of ATM/CHK2 signaling (20, 31). We found that SMG8/9 mutant cells displayed a much-reduced ATM/CHK2 response to ATRi exposure, when assessed by ATM^T1981^ and CHK2^T68^ phosphorylation (Fig. 4M), suggesting that the need to buffer ATR inhibition via ATM activation was minimized. Considering these data together with our prior cell-cycle observations and the ATRi resistance phenotype, it is reasonable to conclude that that *SMG8* or *SMG9* mutations cause ATRi resistance by preventing the DNA damage/RFS caused by ATRi and/or by enhancing the repair of DNA damage.

We used DNA fibre analysis to formally estimate RFS in WT and SMG8/9 mutant cells. While SMG8/9 mutations did not prevent ATRi-induced replication origin firing (Supplementary Fig. S4F), they did increase replication fork processivity despite exposure to ATRi (Fig. 4N). By evaluating the synchronized progression of sister forks emanating from the same origin (asymmetry of sister forks is a marker of fork stalling), we found that the SMG8 or SMG9 mutant cells displayed significantly lower levels of fork stalling after ATRi exposure compared with WT cells (Fig. 4O). These results were consistent with our previous observations indicating that *SMG8/9* mutations prevent RFS and explain the absence ATRi-induced cell-cycle checkpoint, γH2AX, pRPA, and 53BP1 responses in SMG8/9 mutant cells.

Given the fork speed phenotype of SMG8/9-defective cells, we wondered whether a difference in the abundance of endogenous impediments to replication fork progression could explain this increased replication processivity. One reasonable explanation could be a difference in the abundance of RNA:DNA hybrids (R loops) in SMG8/9-defective cells, as aberrant R loops are known to cause genomic instability and have been previously described to activate the ATR/CHK1 pathway (48, 49). Using an antibody that specifically recognizes R loops (S9.6 antibody), we found that ATRi elicited a similar increase in R loop burden in both WT and SMG8/9-defective cells (Supplementary Fig. S4G). This suggested that SMG8/9 defects were unlikely to reduce ATRi sensitivity by reducing either the basal level or ATRi-induced burden of R loops.

**Figure 5.**
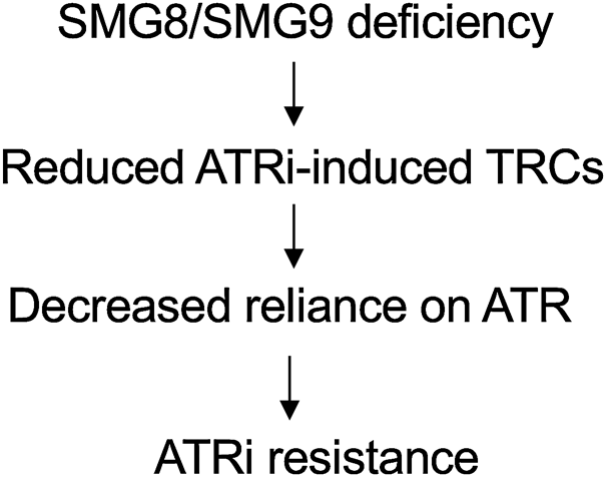
A proposed model of ATRi resistance caused by SMG8/9 loss of function.

To further understand the mechanism by which SMG8/9-defective cells become resistant to ATRi, we considered recent work indicating that ATR protects the genome by suppressing TRCs, that is collisions between RNA polymerase/transcriptional machinery and replication forks (12, 50). In particular, depletion of ARID1A (the YCC6 gastric cancer cell line used here is ARID1A mutant/defective), has been shown to repress RNA polymerase II (RNAPII) transcription (63). On the basis of these observations, we hypothesized that loss of SMG8/9 function could cause ATRi resistance by reducing the burden of TRCs; a reduction in TRCs would in turn reduce the reliance upon ATR and thus relative resistance to ATR inhibition. To test this hypothesis, we assessed whether ATRi elicited the same level of TRCs as in WT cells. Using a previously described proximity ligation assay, which estimates the number of TRCs by quantifying colocalization of RNA polymerase II with the replication-associated protein PCNA (12, 49, 50), we found that ATRi-induced TRCs were decreased in SMG8/9-defective cells compared with WT cells (Fig. 4P). This suggested that SMG8/SMG9 defects cause ATRi resistance by suppressing TRCs; this in turn would likely decrease reliance on ATR, leading to ATRi resistance (Fig. 5).

## Discussion

ATRis are currently being assessed in a number of clinical trials, including gastric cancer (e.g., NCT03641313). Although it is promising that a number of profound and sustained antitumor responses to ATRi have been observed (22, 28, 29, 31), the mechanisms by which ATRi resistance might emerge in the clinic remain unclear. Prior work has suggested that loss of CDC25A can cause ATRi resistance by preventing premature mitotic entry, thus allowing cells to repair the DNA damage/RFS caused or exacerbated by ATRi (32). Here we show that ATRi resistance also emerges via defects in the SMG8/9 heterodimer. This form of drug resistance appears to be SMG1 dependent and distinct from CDC25A-mediated resistance. While CDC25A-mediated resistance is associated with the abolition of mitotic entry (32), ATRi resistance in SMG8/9-defective cells manifests as a phenotype where ATR remains inhibited (CHK1 phosphorylation is still suppressed) but where the RFS, DDR, and cell-cycle effects of ATRi are suppressed (Fig. 4; Supplementary Fig. S4). Furthermore, mutations in SMG8/9 cause a reduction in ATRi-induced TRCs (Fig. 4P), which is increasingly recognized as a driver of ATRi sensitivity (12, 50). A mechanistic model to explain our observations (Fig. 5) is that loss of SMG8/9 function (possibly via elevated SMG1 activity) suppresses the formation of ATRi-induced TRCs or increases their resolution before they exacerbate RFS and become deleterious to the cell. Although we were not able to associate the observed phenotypes with UPF1 function in our models, how such a mechanism might be related to the role of SMG8/9/1 and UPF1 in NMD, or whether this is independent of NMD remains to be established. Furthermore, while we cannot exclude the possibility that SMG1 is upregulated in SMG8/9-deficient cells as a consequence of NMD dysregulation (NMD factors, including SMG1, are known NMD targets themselves; refs. 64, 65), it should be noted that although the SURF complex is classically associated with NMD, mutations in SMG8/9 are not necessarily associated with altered premature truncating mutation NMD (66, 67).

Precisely how SMG8/9 defects minimize ATRi-induced TRCs and RFS remains to be determined, although this does not appear to be by lowering the R loop burden of cells (Supplementary Fig. S4G). One plausible explanation would be that R loops are not the only cause of TRCs; other causes include DNA supercoiling and/or torsional stress (e.g., via malfunction in the topoisomerase machinery), or the appearance of non-B DNA structures (hairpins, triplex DNA, or G-quadruplexes) in the genome (68). In addition, not all TRCs cause an increase in R loops (49): head-on TRCs, when the direction of replication and transcriptional machinery in TRCs is in opposition, cause an increase in R loops, but codirectional TRCs, when the replication and transcriptional machinery are in the same orientation, do not (49). Finally, previous studies investigating other human cancer cell models describe how ATRi-induced replication stress is not directly or solely influenced by DNA:RNA hybrid level but also by other events (i.e., number of origin firings, transcription dysregulation) that have an impact in TRC levels, which are directly related to ATRi response (12, 50).

In terms of the translational implications of our work, we propose that SMG8, SMG9, and SMG1 should be assessed as candidate biomarkers of ATRi resistance, in addition to CDC25A and its paralogs. We are currently optimizing clinical grade IHC assays (69) and DNA/RNA sequencing panel approaches to enable these candidate biomarkers to be assessed in prospective clinical studies evaluating ATRi in gastric cancer.

## Supplementary Material

Supplementary Data

Supplementary Table
