# Supplementary material for: SMG8/SMG9 Heterodimer Loss Modulates SMG1 Kinase to Drive ATR Inhibitor Resistance
Source: Cancer Res. 2022 Oct 23;82(21):3962–73. doi: 10.1158/0008-5472.CAN-21-4339 (PMC9627126; doi:10.1158/0008-5472.CAN-21-4339)
Supplement: Supplementary Data [file can-21-4339_supplementary_data_suppsd.docx]

**Supplementary Information Index:**

1- Supplementary Figures and Figure legends

2- Supplementary Materials and Methods (and associated references)

3- Supplementary Tables 1 and 2

4- Uncropped Western Blot Figures

**1- Supplementary Figures**

**Supplementary Figure 1 (related to figure 1) A.** ARID1A immunohistochemistry of the HCT116 WT and **B.** HCT116 ARID1A -/- cell lines, used as controls. **C.** ARID1A protein expression in GC PDXs. ARID1A immunohistochemistry shows that four of seven GC PDXs are ARID1A deficient (PDX1-4). PDX5-7 are ARID1A proficient. **D.** ARID1A protein expression in GC PDXs by western blotting. **E-H.** ARID1A deficient GC PDXs are sensitive to M4344 ATR small molecule inhibition with PDX1, PDX2 and PDX4 exhibiting a dose response. **I-K.** ARID1A proficient GC PDXs are also sensitive to M4344 ATRi at a lower extent. GC PDX7, an ATM deficient model, displays the most profound sensitivity. The dotted line indicates the duration of ATRi exposure (also illustrated by the M4344 blue arrow). **L.** ATM protein expression in GC PDXs by western blotting. **M.** ATM Immunohistochemistry H-scores for the seven PDX models used show a complete loss of *ATM* expression in the ATRi sensitive model PDX7. **N.** Examples of ATM immunohistochemistry staining in ATM deficient PDX7, compared to ATM proficient PDX6. **O.** Z-scores of essential and non-essential genes in our screen indicating a statistically significant reduction in the number of cells containing sgRNA targeting essential genes after ATRi treatment (unpaired Wilcoxon rank sum test p value). **P.** Scatter plots representing common ATRi resistance genes in Wang *et* al. for MCF10A **Q.** HCT116 and **R.** HEK293A cell lines. Western blots in this figure are representative of three or more biological replicates.

**Supplementary Figure 2 (Related to Figure 2). A,B** Diagram of mutations in the YCC6 SMG8 and SMG9 mutant (Mut) clones, compared to the WT sequence as determined by Sanger sequencing. **C,D** ATRi dose response curves (384 well plate, 5-day assay) illustrating no increased sensitivity to berzosertib or AZD6738 in the WT YCC6 cells (Black) or the SMG8 Mut 1 cells (Blue) when *GPF* cDNA expression was induced by doxycycline exposure (dotted lines) compared to the doxycycline negative arm (continuous lines) **E,F** Diagram of mutations in the SMG8 and SMG9 HAP1 isogenic clones, compared to the WT sequence determined by Sanger sequencing. **G.** Western blot showing SMG8 loss of protein expression in a HAP1 SMG8 Mut clone. **H.** Immunoprecipitation showing SMG9 loss of protein expression in a HAP1 SMG9 Mut clone. **I**. ATRi dose response curves (384 well plate, 5-day assay) in SMG8 (blue) and SMG9 (red) HAP1 Mut clones. SMG8 and SMG9 HAP1 mutant clones are more resistant to AZD6738 compared with negative control cells transduced with a non-targeting gRNA. **J**. Western blot showing SMG8 loss of protein expression in the HCT116 SMG8 knock-down cells. **K,L.** ATRi dose response curves (384 well plate, 5-day assay) in the HCT116 SMG8 and siCON2 knock-down cells. HCT 116 SMG8 knock-down cells are statistically more resistant to both berzosertib and AZD6738 compared with negative control cells transfected with a non-targeting siRNA. **M**. Western blot showing SMG9 loss of protein expression in the HCT116 SMG9 knock-down cells. **N,O**. ATRi drug response survival curves (384 well plate, 5-day assay) in the HCT116 SMG9 and siCON2 knock-down cells. HCT 116 SMG9 knock-down cells are statistically more resistant to both berzosertib and AZD6738 compared with negative control cells transfected with a non-targeting siRNA.

**Supplementary Figure 3 (Related to figure 3). A,B.** SMG1 knock down experiment (5nM siRNA, 384 well-plates) showing the normalised percent of inhibition of the SMG8 Mut 1 isogenic clone (red) or SMG9 Mut 2 isogenic clone (red) compared with the WT cells (blue) following exposure to four single siSMG1 siRNAs (siSMG1-05 to 08; negative control: siCON2; positive control: siPLK1). **C.** Western blot showing phospho UPF1 (Ser1127) and UPF1 expression in the WT, SMG8 Mut 1 and SMG9 Mut 2 cells. SMG8 and SMG9 Mut cells show a mild increase in phospho-UPF1 expression, compared to the WT. **D.** UPF1 knock-down western blot showing UPF1 silencing in the WT, SMG8 Mut 1, SMG9 Mut 2 cells, compared to the siCON2 exposed cells after 72hr of 20nM siRNA exposure. **E,F.** berzosertib drug curves comparing WT (blue) and SMG8 Mut 1 (red) cells or the SMG9 Mut 2 cells (red) (384 well plate, 5-day assay) after transfection with siCON2 or siUPF1 showing increased resistance in the WT cells when UPF1 is knocked down and no significant difference in ATRi response in the SMG8 and SMG9 Mut cells**. G-I** ATRi drug response survival curves (384 well plate, 5-day assay) in the YCC6 WT cells (blue) SMG8 Mut 1 and SMG9 Mut 1 cells (red) after exposure to four single siUPF1 siRNAs (siUPF1-05 to 08) or siCON2 as a negative control, supporting the results observed with the siUPF1 pool reagent in Supplementary figure 3**F** and 3**G**. Normalised percent of inhibition was calculated from the CellTitre-Glo reads following the next equation ((Mean siCON2-Sample)/(Mean siCON2-mean siPLK1)). All panels in this figure are representative of 3 or more biological replicates.

**Supplementary Figure 4 (related to Figure 4). A.** Western blot showing no difference in CDC25A or CDC25B protein expression in the SMG8 and SMG9 Mut cells compared to the WT, after 48hr of DMSO or 400nM ATRi exposure. **B,C.** berzosertib monotherapy (continuous line) and berzosertib + MK1775 combination (dotted line) dose-response curve comparing the surviving fraction of SMG8 Mut 1 cells (blue) or SMG9 Mut 2 (red) to the WT cells**.** Δ AUC or Area Under the Curve is used to measure the difference in the surviving fraction of cells treated with the combination of the two drugs (ATRi+WEEi) compared to the ATRi monotreatment, which shows no difference in either of the SMG8 or SMG9 Mut clones, compared to the WT. **D.** Immunofluorescent staining for γ-H2AX (red) or DAPI (blue) in cells treated with either HU (3hr of 3mM) or berzosertib (24hr of 400nM or 800nM). SMG8 and SMG9 Mut cells show lower levels of γ-H2AX nuclear staining under ATRi exposure compared to the WT. *P* values were calculated using Mann-Whitney test for non-parametric samples, measuring the relative intensity per nucleus (arbitrary units; Fiji, Image J). **E**. Immunofluorescent staining for 53BP1 (red), Cyclin A2 (green) and DAPI (Blue) in the cells after either HU (3hr of 3mM) or berzosertib (24hr of 400nM or 800nM) exposure. Cyclin A2 positive SMG8 and SMG9 Mut cells show lower levels of 53BP1 foci staining after ATRi exposure compared to the WT. *P* values were calculated using Mann-Whitney test for non-parametric samples, measuring the number of 53BO1 foci per Cycline-A2 positive nucleus using FIJI (Image J).

**F.** Similar percentage of origins scored in the SMG8, SMG9 Mut and WT cells after 24hr of 300nM of ATRi exposure in the DNA fiber experiment in Figures **J** and **K**. Statistics representative of the comparison of the fold change between untreated/treated cells in the SMG8 Mut and SMG9 Mut cells vs the WT. All panels in these figures are representative of 3 or more biological replicates. **G**. Normalised S9.6 mean intensity in the YCC6 WT and SMG8/9 mutant cells after 24hr of ATRi or DMSO exposure show no difference in the induction/resolution of DNA:RNA hybrids in our isogenic models. U2OS cells used as a control. RNASEH1 treatment included to confirm specificity of the S9.6 antibody. P values correspond to Mann-Whitney test for non-parametric samples, comparing >100 cells per biological repeat, in an n=3.

**2- Supplementary Materials and Methods**

**Western blotting**

Whole cell lysates were separated using 3-8% Tris-Acetate gels (Thermo Fisher) or 4%–12% sodium dodecyl sulfate-polyacrylamide gel electrophoresis (SDS-PAGE) Bis-Tris gel, transferred to nitrocellulose membrane, blocked in 5% milk or BSA and blotted with antibodies (**Supplementary Table 2).**

**Polymerase Chain Reaction (PCR) and gel electrophoresis**

Generally, PCR amplicons were generated using 100ng of DNA in 50µl reactions, using the New England BioLabs Q5® High-Fidelity DNA Polymerase kit, according to manufacturer’s instructions, using specific primers (**Supplementary Table 1).** PCR was carried out on a thermocycler as follows: 98°C for 2 minutes, followed by 30 cycles of 98°C for 30 seconds, 60°C (or optimised temperature) for 30 seconds and 72°C for 20 seconds, followed by a final step at 72°C for 2 minutes. Primers were diluted to 10µM for use in each PCR reaction. PCR products were separated by agarose gel electrophoresis by mixing with 6x loading dye (New England Biolabs). DNA was then visualised using an ultraviolet transilluminator (Syngene).

**TOPO cloning and Sanger Sequencing of mutant clones**

PCR purification was carried out using the QIAquick PCR Purification Kit (Qiagen). 100ng purified PCR products were cloned into the pCR-Blunt II-TOPO vector using the Zero Blunt TOPO PCR Cloning kit (Thermo Fisher) following manufacturer’s protocol. The final mix was incubated 1hr at room temperature (RT) and transformed with 150µl of competent cells (DH5α) were mixed gently with 5µl of cloned product. After 30 minutes of incubation on ice, tubes were heated at 42ºC for 45 seconds and cooled back on ice. 300µl of outgrowth SOC media (Thermo FIsher) was added and the tubes were placed in a shaking incubator for 1hr at 37ºC. After incubation, bacteria were streaked out on Petri dishes and incubated overnight at 37ºC. Single colonies were picked and expanded in kanamycin selective media overnight at 37°C. DNA was extracted using the Qiaprep Spin Miniprep/Midiprep kit (Qiagen). For Sanger Sequencing, 15µl of purified DNA was mixed with 2µl of 10µM forward or reverse primers for the target gene sequence and prepared at a final concentration of 100ng/µl in the case of plasmid DNA or 10ng/µl for purified PCR products. Samples were run by Eurofins Genomics (<https://www.eurofinsgenomics.eu)> using specific primers (**Supplementary Table 1)** and results were analysed using the sequence alignment tool in ApE Plasmid Editor (https://jorgensen.biology.utah.edu/wayned/ape/).

**Assessment of ATRi efficacy in Patient-Derived Xenografts**

An *in vivo* efficacy study testing the oral ATR inhibitor, M4344 (Merck), was carried out by Crown Bioscience, in an agreed academic collaboration with Merck Serono. Seven gastric adenocarcinoma PDX models were selected from the Crown Bioscience database according to their *ARID1A* mutational status (exome and RNA sequencing), which was provided by the company and additionally validated in-house. We performed DNA sequencing using the PGM 318 Chip (IonTorrent) using a previously designed panel of genes, including *ARID1A* [66]. Additionally, loss of ARID1A or ATM protein expression was tested by immunohistochemistry on formalin-fixed paraffin-embedded sections across all the tumour samples using specific antibodies and the Dako-Autostainer Link 48 with the EnVision FLEX kit as per manufacturer’s instructions (Agilent Technologies). HCT116 ARID1A WT and deficient cells were used as positive and negative controls for ARID1A expression respectively; and two independent pathologists, who were blinded to the mutational status of the samples, analysed the sections. Although single agent M4344 delivered at 20mg/kg daily is largely well tolerated in other *in vivo* tumour models, an additional single agent M4344 arm at a reduced dose of 10mg/kg daily was included as a precaution in the event that tolerability issues are experienced in the mice. Seven randomly distributed female BALB/c nude mice, bearing tumours with an approximate size of 100-200mm^3^, received the following treatments by oral gavage: M4344 20mg/kg twice a day (BID) for 2 days a week; M4344 10mg/kg once a day (SID) for 2 days a week; M4344 3mg/kg daily or vehicle control. The initial PDX (PDX1) was used to optimise the dose and subsequent groups were treated at a dose of 10mg/kg twice a week or 5 mg/kg M4344 daily. Sample sizes for treatment groups were calculated based on the Cohen principle (1998), considering the following parameters: effect on 75% are measured as significant, assuming normal distribution, *p* value (α) <0.05 and a ß of 0.95. Calculation of the variance is assumed to be about 40% for a randomised population (tumours are randomised to have equally distributed standard variations within the treatment groups).

**SMG8 and SMG9 CRISPR/Cas9 mutagenesis**

60% confluent cells were transfected in 6-well plates, following a standard forward transfection protocol using 2500ng Geneart Platinium Cas9 nuclease (Thermo Fisher) per well, 5µl of Lipofectamine Cas9 Plus Reagent, 7.5µl Lipofectamine CRISPRMAX Reagent (Thermo Fisher) and 650ng of the sgRNA. A sgRNA with no homology to any known mammalian gene was used as a negative control. Single cells were sorted into 96-well plates using a FACS Aria (Becton Dickinson) and incubated at 37°C until colonies expanded. Colonies were harvested and tested for SMG8 and SMG9 protein expression by western blot or IP, respectively, and for mutations, PCR and Sanger sequencing using the U6 and Scaf primer sequences, amplifying the region surrounding the sgRNA was used. **Supplementary Table 1** contains all the primers used in this paper.

**CRISPR screen next-generation sequencing data analysis**

MAGeCK (Model-based Analysis of Genome-wide CRISPR/Cas9 Knockout) software was used to generate sgRNA counts according to the sequences present in the genome-wide CRISPR library [1]. Using normalised read count data from MAGeCK, quality checks were performed (distribution of read counts, clustering of samples), to confirm the robustness of the data. For downstream analysis of sgRNA read count data, two approaches were used for comparative analysis: MAGeCK and z-score. From MAGeCK workflow, we extracted a ranked list of positively selected hits generated using its robust ranking aggregation algorithm (RRA) approach [1]. For the z-score analysis approach, the low abundant guides with a read count of zero in the T=0 sample were first identified and removed from the analysis. Then, raw read counts were converted to parts per ten million (pptm) counts to account for variation in the amount of DNA sequenced. The raw pptm counts were log2 transformed (after adding a pseudo count of 0.5) before calculating the viability effect (VE) z-scores (see equation 1). We define VE as the rate of decrease in abundance of each sgRNA in the population over time in the absence of drug treatment (i.e., in the DMSO sample),

$Viability Effect (VE)=\frac{(\mathrm{Tn}_{DMSO} - T0) - median (\mathrm{Tn}_{DMSO}- T0)}{MAD(\mathrm{Tn}_{DMSO} -T0)}$ (1)

where Tn represents the log2 pptm counts of DMSO sample at time point *n* and T0 represents the log2 pptm counts of sample *at* time point 0*;* MAD is the median absolute deviation.

We considered a threshold of z-score > 2 for resistant sgRNAs and z-score < -2 for sensitizing sgRNAs. A z-score was assigned to each gene by taking the median z-score of sgRNAs targeting a particular gene. Ranks for positive selection were generated by sorting results based on their z-score in descending order. A final list of hits was consolidated from both z-scores and MAGeCK approaches by taking the rank product of their individual ranks (**Supplementary Table 3**).

**Methods References**

1. Li, W., et al., *MAGeCK enables robust identification of essential genes from genome-scale CRISPR/Cas9 knockout screens.* Genome Biol, 2014. **15**(12): p. 554.

2. Chong, I.Y., et al., *Mapping genetic vulnerabilities reveals BTK as a novel therapeutic target in oesophageal cancer.* Gut, 2018. **67**(10): p. 1780-1792.

**3- Supplementary Tables 1 and 2**

**Supplementary Table 1. PCR primers, sgRNAs and siRNAs used in this study.**

| **Reagent** | **Sequence (5’>3’)** | **Application** | **Company** |
| --- | --- | --- | --- |
| SMG8.1 F | CACCAATACGGTGTGCGACCGAC | PCR & Sequencing Primer | IDT |
| SMG8.1 R | AAACGTCGGTCGCACACCGTATT | PCR & Sequencing Primer | IDT |
| SMG8.2 F | CACCGGTCCAGCAACATACCTAC | PCR & Sequencing Primer | IDT |
| SMG8.2 R | AAACGTAGGTATGTTGCTGGACC | PCR & Sequencing Primer | IDT |
| SMG9.1 F | CACCGAACGATGGGCTTCTCCAG | PCR & Sequencing Primer | IDT |
| SMG9.1 R | AAACCTGGAGAAGCCCATCGTTC | PCR & Sequencing Primer | IDT |
| SMG9.2 F | CACCGGACCCCTCGGTACCTCGA | PCR & Sequencing Primer | IDT |
| SMG9.2 R | AAACTCGAGGTACCGAGGGGTCC | PCR & Sequencing Primer | IDT |
| U6-F | GGCCTATTTCCCATGATTCCTTC | PCR & Sequencing Primer | IDT |
| Scaf-R | ACTCGGTGCCACTTTTTCAA | PCR & Sequencing Primer | IDT |
| SMG8.1 | AATACGGTGTGCGACCGAC | sgRNA for CRISPR editing | Horizon discovery |
| SMG8.2 | GGTCCAGCAACATACCTAC | sgRNA for CRISPR editing | Horizon discovery |
| SMG9.1 | GAACGATGGGCTTCTCCAG | sgRNA for CRISPR editing | Horizon discovery |
| SMG9.2 | GGACCCCTCGGTACCTCGA | sgRNA for CRISPR editing | Horizon discovery |
| siSMG8 | SMART pool D-016480 | siRNA silencing | Horizon discovery |
| siSMG9 | SMART pool D-020561 | siRNA silencing | Horizon discovery |
| siSMG1 | SMART pool D-005033 | siRNA silencing | Horizon discovery |
| siUPF1 | SMART pool D-011763 | siRNA silencing | Horizon discovery |
| siPLK | SMART pool D-003290 | siRNA silencing | Horizon discovery |
| ATM | SMART pool D-003201 | siRNA silencing | Horizon discovery |
| siCON2 | SMART pool D-001206 | siRNA silencing | Horizon discovery |
| siCON1 | SMART pool D-001810 | siRNA silencing | Horizon discovery |
|  |  |  |  |

Abbreviations: F, forward; R, reverse; IDT, integrated DNA technologies.

**Supplementary Table 2. Antibodies used in this study**

| Protein | Antibody dilution | Experi-ment | Species | Company | Product code |
| --- | --- | --- | --- | --- | --- |
| ARID1A | 1:1000 | WB | Rabbit | CST | 12354 |
| ß-Actin | 1:2000 | WB | Mouse | CST | 3700 |
| phospho CHK1 (S345) | 1:500 | WB | Rabbit | CST | 23485 |
| phospho CHK1 (S317) | 1:500 | WB | Rabbit | CST | 12302 |
| SMG8 | 1:3000 | WB | Rabbit | Abcam | Ab80472 |
| SMG9 | 1:2000 | IP/WB | Rabbit | Bethyl | A302-211A |
| CHK1 | 1:1000 | WB | Mouse | CST | 2360 |
| phospho CHK2 (T68) | 1:1000 | WB | Rabbit | CST | 2197 |
| CHK2 | 1:2000 | WB | Rabbit | CST | 6334 |
| phospho ATM (S1981) | 1:1000 | WB | Rabbit | Abcam | Ab81292 |
| ATM (D2C2) | 1:2000 | WB | Rabbit | CST | 2873 |
| SMG1 | 1:500 | WB | Rabbit | CST | 9149 |
| CDC25B | 1:1000 | WB | Rabbit | CST | 9525 |
| CDC25A | 1:500 | WB | Rabbit | CST | 3652 |
| phospho HistoneH3 | 1:1000 | WB | Mouse | Millipore | 05-806 |
| phospho RPA32 (S4/S8) | 1:2000 | WB | Rabbit | Bethyl | A300-245A |
| RPA32 | 1:4000 | WB | Rabbit | Bethyl | A300-244A |
| γH2AX | 1:2000 | WB | Rabbit | CST | 2577 |
| GFP | 1:4000 | WB | Mouse | Roche | 11814460001 |
| HA tag | 1:5000 | WB | Mouse | Millipore | 05-904 |
| phospho UPF1 (S1127) | 1:500 | WB | Rabbit | Millipore | 07-1016 |
| UPF1 | 1:5000 | WB | Rabbit | CST | 12040 |
| Vinculin | 1:5000 | WB | Mouse | Santa Cruz | Sc-73614 |
| CRISPR-Cas9 | 1:2000 | WB | Mouse | Diagenode | C15200203-100 |
| 53BP1 | 1:1000 | IF | Mouse | Millipore | MAB3802 |
| γ-H2AX | 1:500 | IF | Mouse | Millipore | 05-636 |
| Cyclin A2 | 1:500 | IF | Rabbit | Abcam | Ab181591 |
| Anti-Mouse, Alexa fluor 555 | 1:400 | IF | Donkey | Thermo Fisher | A31570 |
| Anti-Rabbit Alexa fluor 488 | 1:200 | IF | Donkey | Thermo Fisher | A21206 |
| IRDye 800CW anti-Rabbit IgG | 1:10000 | WB | Donkey | LI-COR | 926-32213 |
| IRDye 800CW anti-Mouse IgG | 1:10000 | WB | Goat | LI-COR | 925-32210 |
| Anti-Mouse IgG | 1:5000 | WB |  | CST | 7076 |
| Anti-Rabbit IgG | 1:5000 | WB |  | CST | 7074 |
| S9.6 | 1:500 | IF | Mouse | Kerafast | ENH001 |
| PCNA (PC-10) | 1:500 | IF | Mouse | Santa Cruz | Sc56 |
| phospho RNA Pol II (Ser2) | 1:1000 | IF | Rabbit | NB | NB1001805 |

Abbreviations: WB, western blot; IP, immunoprecipitation; IF, immunofluorescence; CST, cell signalling technology; NB, Novus Biologicals.

**4- Uncropped Western Blot Figures**
